# Mechanical Vibration Damping and Compression Properties of a Lattice Structure

**DOI:** 10.3390/ma14061502

**Published:** 2021-03-18

**Authors:** Katarina Monkova, Martin Vasina, Milan Zaludek, Peter Pavol Monka, Jozef Tkac

**Affiliations:** 1 Faculty of Manufacturing Technologies, Technical University in Kosice, 080 01 Presov, Slovakia; peter.pavol.monka@tuke.sk (P.P.M.); jozef.tkac@tuke.sk (J.T.); 2 Faculty of Technology, Tomas Bata University in Zlin, Nam. T.G. Masaryka 275, 760 01 Zlin, Czech Republic; zaludek@utb.cz; 3 Faculty of Mechanical Engineering, VSB-Technical University of Ostrava, 17. Listopadu 15/2172, 708 33 Ostrava-Poruba, Czech Republic

**Keywords:** mechanical vibration, displacement transmissibility, excitation frequency, compression behavior, Acrylonitrile Butadiene Styrene, 3D printing

## Abstract

The development of additive technology has made it possible to produce metamaterials with a regularly recurring structure, the properties of which can be controlled, predicted, and purposefully implemented into the core of components used in various industries. Therefore, knowing the properties and behavior of these structures is a very important aspect in their application in real practice from the aspects of safety and operational reliability. This article deals with the effect of cell size and volume ratio of a body-centered cubic (BCC) lattice structure made from Acrylonitrile Butadiene Styrene (ABS) plastic on mechanical vibration damping and compression properties. The samples were produced in three sizes of a basic cell and three volume ratios by the fused deposition modeling (FDM) technique. Vibration damping properties of the tested 3D-printed ABS samples were investigated under harmonic excitation at three employed inertial masses. The metamaterial behavior and response under compressive loading were studied under a uniaxial full range (up to failure) quasi-static compression test. Based on the experimental data, a correlation between the investigated ABS samples’ stiffness evaluated through both compressive stress and mechanical vibration damping can be found.

## 1. Introduction

Nowadays, ecological problems and efforts to save energy are of great importance in industry. Industrial processes’ design can be improved to overcome these problems, but these design improvements have inevitable limitations. Further process improvements can be made by developing and improving the materials used in the processes. Design will inevitably change as new materials are used. Currently, an increasingly important characteristic for materials used in various fields, such as automotive, aerospace, construction, and biomedical industries, is being lightweight, usually caused by material porosity. Weight reduction can significantly reduce material and energy consumption, carbon dioxide emission, and waste generation, promoting the sustainability of materials used in various industries. Porous materials with good mechanical properties can reduce production costs, make production more efficient, and make a product more environmentally friendly.

The development of additive technologies now makes it possible to produce lightweight metallic and nonmetallic porous materials with properties that are highly controllable. This means that their structure is designed in a controlled way. Such a structure is based on a so-called basic cell, which is regularly distributed (or according to a certain rule) in the core of the product and whose size is also explicitly controlled. A special category of cellular materials is materials with a lattice structure. In the language of structural engineering, a lattice truss or spatial truss is an arrangement of struts that are pin-jointed or rigidly connected at their joints [1]. The investigation of the behavior of cellular materials with lattice structures make it possible to use the most suitable structure (with specified properties) for a component that is particularly stressed in real operation.

The basic building unit of cellular materials is a so-called cell that regularly repeats in the structure and can be distributed in one or more independent directions. The basic cell type can be different from a cubic, from a honeycomb up to a spherical type. Another important property is the specific volume of the material or so-called volume ratio *V_r_*, which is expressed by Equation (1):

(1) $$V_{r}=\frac{Vso}{Vto}$$

where *Vso* is the volume of a solid material used for the cell structure building and *Vto* is the total volume of the sample.

Many researchers have dealt with the mechanical properties of lattice materials. The influence of the length and orientation of the strut on the elastic mechanical response of the modified cubic lattice structures has been studied by Hasanain [2], Tahseen [3], and Alwattar [4]. The reference models’ samples were made by fusion deposition (FDM) technique using acrylonitrile-butadiene-styrene (ABS) material and tested under compression load. It has been observed that the fixed length of a strut of the body-centered cubic (BCC) lattice structure with an angle of 100° offers the highest modulus of elasticity. However, the highest specific deformation energy absorption and specific stiffness, as well as the smallest weight value, were given by the variant strut length with a strut angle of 40°. The results obtained from the finite element analysis (FEA) were compared with experimentally measured data, and a good agreement within the linear elastic limit has been achieved. The experimental tests performed by Iyibilgin [5] to evaluate the compressive properties (yield strength and compressive modulus) of different ABS 3D-printed cellular lattice structures showed that the differences between the different lattice structures were in the range of 7%. Fadeel [6] also discussed the behavior of ABS 3D-printed lattice structures during compression deformation through both physical tests and computer modeling. The results have shown that the strength of the lattice structures is greater when vertical elements are present, and it depends on the lattice geometry rather than its mass. Gautam [7] has been interested in the compressive properties of additively fabricated functionally graded Kagome structures made of Ti–6Al–4V and ABS materials. It was found that a functionally graded Kagome structure provides 35% more energy absorption than the uniform density structure.

Many researchers have also studied the failure of compression-loaded lattice structures. Cantrell [8] and Al Rifaie [9] focused on the failure behavior of 3D-printed lattice structures. They concluded that ABS material exhibited a hybrid behavior between strain and bend dominated properties. The results also showed that the failure of a compressed lattice structure is initiated in the regions of the connection nodes and that the selective placement of vertical support struts in the unit cell affects both the absolute and specific mechanical properties of the lattice structures. Calise and Saigal [10] studied the anisotropy and failure in octahedral lattice structures of two different relative densities, which were made from ABS material using FDM technology. It was found not only that the stiffness and strength of the as-fabricated parts are anisotropic, but also that they are a function of the relative density/strut diameter of the structure.

Several authors have already discussed the stiffness and strength of the various lattice structures [11,12,13,14,15]. Mahshid et al. [16] fabricated structure samples, including solid, hollow, and lattice structure, and compared the strength of structures by the compression test. The result indicated that, though the strength of the lattice structure was lower than that of the solid structure, it can still meet the application requirements, and reduced the use of materials, achieving a lightweight design. Utomo [17] was concerned with the determination of the compressive strength of a 3D polymeric lattice structure as a template in powder metallurgy. Lattice structured samples were made of ABS (Acrylonitrile Butadiene Styrene), PLA (Polylactic Acid), and PVA (Polyvinyl Alcohol) filaments. They were designed in a cubic shape with uniform total dimensions, but with variations in pore size. Both computational and experimental tests were performed to determine the compressive strength of these structures, while observing the resulting stresses, strains, and deformations of the 3D polymer lattice. The results confirmed that the variations in materials and pore size significantly affect the stress, strain, and deformation of the 3D polymer lattice materials. Ozdemir et al. [18] carried out a quasi-static test analysis on lattice structures with cubic cells, diamond cells, and re-entrant cells, respectively. They studied the dynamic load deformation behavior of a lattice structure, including the failure process and stress–strain response. Under the condition of a low-speed and high-speed collision, the lattice structures showed a noticeable difference in deformation behavior and energy absorption effect.

Some researchers evaluated the stiffness of a structure not only by mechanical compression, but also by frequency analysis. Azmi, A. S. et al. [19] studied the effect of the size of the strut radius on the natural frequencies of the ABS lattice structure, and the results showed that a larger size of the strut’s radius would lead to higher natural frequencies. Simsek et al. [20] were interested in 3D-printed porous sandwich structures for resonance frequency applications. In this study, the frequency response predictions of a finite element-based model of the gyroid sandwich structure were first validated in terms of its natural frequencies and mode shapes, while the effects of plate and gyroid wall thickness on dynamic properties were investigated. Tyburec [21] examined a convex linear semidefinite programming formulation for truss topology optimization to design an efficient non-uniform lattice as an internal structure. The internal structure not only reduces the effect of wall instabilities, reflected in the increase of fundamental natural frequency of free-vibration, but also keeps the weight low, ensures the manufacturability with conventional three-dimensional printers, and withstands the stresses during the production process. The results confirmed that the 3D-printed optimized internal structure nearly doubles the fundamental natural frequency of free-vibration, and thus increases the working frequency of the machine tool.

According to Pantazopoulos [22], if the loading conditions and the maximum (unprotected) crack or minimum (detectable) crack size are known, the minimum fracture strength (toughness) of the material can also be determined, and the information can be used for material selection during the design stage. Experimental testing of material properties is thus important not only for component design, but also for defining the boundary conditions of numerical analysis.

The aim of the research is to investigate the influence of the basic cell size and volume ratio on the vibration damping and compression behavior of a basic lattice structure fabricated from material ABS to use it in the most suitable applications for the parts of daily use or in engineering practice. Despite many studies conducted in the field of cellular metamaterials, to the best of the authors’ knowledge, the study of the combination of selected factors (i.e., ABS material, volume ratio *V_r_*, and basic cell size *a*) on the investigated properties (i.e., compression and vibration damping behavior) of the BCC lattice structure has not been considered in any research.

## 2. Materials and Methods

### 2.1. Characteristics of Samples

The performance of a component is limited by the specific properties of the materials from which it is made. This means that, to achieve a desired level of performance, the values of the design-limiting properties must meet certain targets, and those that fail to do so are not suitable. On the other hand, a special material can give the product an extraordinary combination of properties that increases its production efficiency from the view of its usage in technical practice and in terms of production costs. Such materials also belong to porous materials based on lattice structures applied in the core of the final component, also making it lighter with less material consumption.

**Figure 1:**
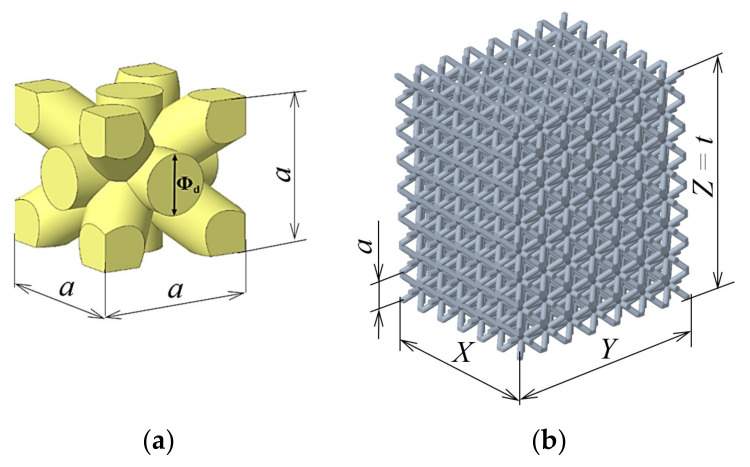
Lattice structure: (**a**) basic body-centered cubic (BCC) cell and (**b**) virtual model of the lattice sample.

To investigate the relationship between mechanical vibration and pressure properties, a simple BCC (body-centered cubic) cell with cylindrical struts was chosen (Figure 1a). This cell was regularly “distributed” in all three directions, *x, y,* and *z*, by shifting by the entire cell size. Within the research, the samples were made with three basic cell sizes *a* = 5, 7, and 10 mm. The dimensions of the individual experimental specimens were controlled by the relation *X* × *Y* × *Z* = 6*a* × 7*a* × 8*a*, which means that the samples with the cell size *a* = 5 mm had dimensions of 30 mm × 35 mm × 40 mm, samples with the cell size *a* = 7 mm had dimensions of 42 mm × 49 mm × 56 mm, and samples with the cell size *a* = 10 mm had dimensions of 60 mm × 70 mm × 80 mm.

The building direction in 3D-printing corresponded to the *z*-axis. This dimension in the *z*-axis direction can also be considered as the thickness of the material whose damping properties were examined and, therefore, this dimension was referred to as “*t*” in the vibration tests; see Figure 1b.

**Figure 2:**
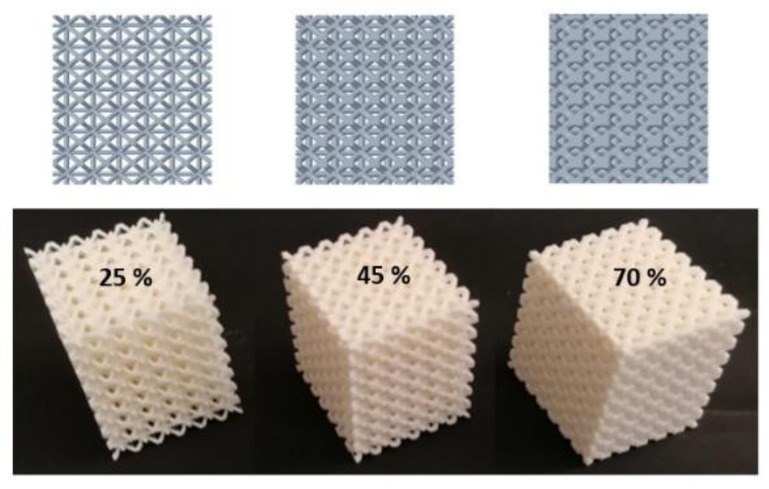
3D models and produced samples with the cell size *a* = 5 mm and all three volume ratios *V_r_* = 25%, 45%, and 70%.

Furthermore, samples were prepared for each base cell size with three different volume ratios *V_r_* = 25%, 45%, and 70%, which were driven by changing the strut diameter Φ_d_ (see Figure 1a). It was varied from Φ_d_ = 1 mm (sample with *a* = 5 mm and *V_r_* = 25%) to Φ_d_ = 4 mm (sample with *a* = 10 mm and *V_r_* = 70%). Five samples of each type (of the same size *a* and volume ratio *V_r_*) were produced to repeat the vibration damping and compression tests and were evaluated statistically so that a total of 45 samples were produced. The top view on 3D models generated in software PTC Creo (version 6, PTC Inc., Boston, MA, SUA). and produced samples with the cell size *a* = 5 mm and all three volume ratios *V_r_* = 25%, 45%, and 70% are presented in Figure 2.

**Figure 3:**
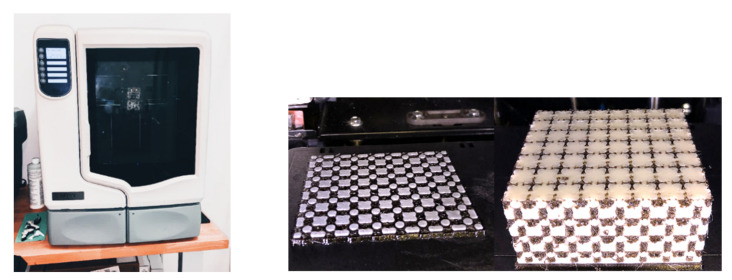
The 3D printer uPrint SE (**left**) and two views of the sample in the printing process (**right**).

The samples were produced by the FDM (fused deposition modeling) technique employing the 3D printer uPrint SE (Computer Aided Technology, Buffalo Grove, IL, USA), which uses the soluble supports technology (SST). It is a water-based solution that allows to simply wash away the support material used in the 3D printing process of a component. The part is left smooth and clean with the fine details intact in that case. After removing the sample from the printer, the sample was placed in a cleaning device, where the support material was removed (dissolved) by flowing in the aqueous solution at 70 °C. Because the samples were complex in shape and contained a relatively large amount of supporting material, it was necessary to add to the hot water a solution of SR-100 containing small particles of so-called dust, which ensures faster solubility of the support material. The layer thickness was 0.254 mm. During sample production, all other process parameters were retained as set by the 3D printer manufacturer for ABS material because the machine does not allow the settings to be changed. The 3D printer uPrint SE and two views of the sample in the printing process are shown in Figure 3, where it is possible to see the alternating layering of the basic and support material (black colored).

For the presented research, the plastic material Acrylonitrile Butadiene Styrene (ABS) (Smart Materials 3D, Alcala la Real, Spain) in 3D-printed form was chosen for its properties and wide applicability, good availability, and reasonable price to produce the samples.

All samples within the research were made of ABSplus-P430 Ivory material, and P400SR (Stratasys^®^ Inc., Minneapolis, MN, USA) was used as support material. The basic properties of the ABSplus-P430 Ivory material are listed in Table 1 [23].

### 2.2. Measurement Methodology

#### 2.2.1. Mechanical Vibration Damping Testing

Vibration damping properties of the tested 3D-printed ABS samples were investigated under harmonic excitation of a linear viscously damped single-degree-of-freedom (SDOF) system, which is characterized by the displacement transmissibility *T_d_* (−) as follows [24,25,26,27]:

(2) $$T_{d}=\frac{x_{O}}{x_{I}}=\sqrt{\frac{k^{2}+{\left(c\omega \right)}^{2}}{{\left(k-m\omega^{2}\right)}^{2}+{\left(c\omega \right)}^{2}}}=\sqrt{\frac{1+{\left(2\zeta r\right)}^{2}}{{\left(1-r^{2}\right)}^{2}+{\left(2\zeta r\right)}^{2}}}$$

where *x* is the displacement amplitude on output (*O*) or input (*I*) sides of the tested sample, *k* is the material stiffness (N/m), *c* is the viscous damping coefficient (Ns/m), *ω* is the circular frequency of oscillation (rad/s), *m* is the mass (kg), *ζ* is the damping ratio (−), and *r* is the frequency ratio (−). Depending on the value of the displacement transmissibility, there are three types of mechanical vibrations, namely, resonance (*T_d_* > 1), undamped (*T_d_* = 1), and damped (*T_d_* < 1) vibrations.

The damping and frequency ratios are defined by the following equations [28,29]:

(3) $$\zeta =\frac{c}{2\sqrt{km}}=\frac{c}{2m\omega_{n}}$$

(4) $$r=\frac{\omega }{\omega_{n}}=\frac{\omega }{\sqrt{k/m}}$$

where *ω_n_* is the natural frequency (rad/s), which is proportional to the square root of the ratio of the material stiffness to the mass [30]. Under the condition *dT_d_*/*dζ* = 0 in Equation (2), it is possible to find the frequency ratio *r_m_*:

(5) $$r_{m}=\frac{\omega_{R}}{\omega_{n}}=\frac{2\pi f_{R}}{\omega_{n}}=\frac{\sqrt{\sqrt{1+8\zeta^{2}}-1}}{2\zeta }$$

where *ω_R_* is the circular frequency (rad/s), at which the displacement transmissibility reaches its maximum value [24,27,30], and *f_R_* (Hz) is the resonance frequency. This frequency is always less than the natural frequency *ω_n_* [30]. From Equation (5), it is clear that higher values of the damping ratio *ζ* generally lead to a lower value of the frequency ratio *r_m_* [31,32,33].

**Figure 4:**
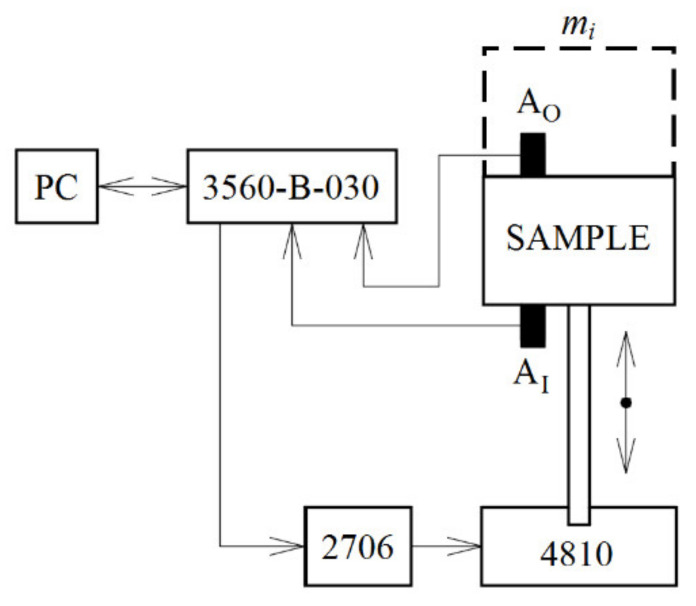
Schematic diagram of the experimental setup for measuring the displacement transmissibility of a linear single-degree-of-freedom system.

Experimental measurements of the displacement transmissibility of the investigated 3D-printed open-porous ABS materials were performed using harmonically excited vibrations in the frequency range of 2−3000 Hz. The schematic diagram of the measuring device for measuring the vibration damping properties of a linear single-degree-of-freedom (SDOF) system are shown in Figure 4. The measuring device consisted of a mini-shaker (BK 4810), a dynamic signal PULSE multi-analyzer (BK 3560-B-030) (Brüel & Kjær, Nærum, Denmark), and a power amplifier (BK 2706) (Brüel & Kjær, Nærum, Denmark). The mini-shaker type 4810 is a compact, permanent magnet, electrodynamic exciter with a maximum force ratio of 10 N. In the case of the harmonically excited mechanical vibrations, it is possible to modify Equation (2) as follows:

(6) $$T_{d}=\frac{a_{O}}{a_{I}}$$

where *a* is the acceleration amplitude on the output (*O*) or input (*I*) sides of the tested sample. The displacement transmissibility was determined from Equation (6) based on the acceleration amplitudes recorded using the BK 4393 piezoelectric accelerometers *A_O_* and *A_I_* (Brüel & Kjær, Nærum, Denmark). Frequency dependencies of the displacement transmissibility of the investigated ABS samples were evaluated depending on the sample thickness (i.e., 40, 56, and 80 mm corresponding to the *z*-axis direction, in which the samples were built), its volume ratio (i.e., 25%, 45%, and 70%), and the inertial mass *m_i_* (i.e., 0, 90, and 500 g) located on the upper side of the harmonically loaded tested samples, as shown in Figure 4. During the experimental measurements of the displacement transmissibility, the tested sample and the inertial mass were rigidly connected. Each measurement was repeated five times at an ambient temperature of 23 °C.

#### 2.2.2. Compression Testing

Compression testing is performed as part of the design process, in the production environment, or in the quality control laboratory, and it is used to assess the strength of components, e.g., automotive, aeronautical, civil, or mechanical engineering industries, to characterize the compressive properties except for other materials, including porous materials, or to evaluate the performance of products [34].

The main purpose of compression testing in the research was to determine the behavior and response of the metamaterial under compressive loading by measuring fundamental variables such as stress and strain. In combination with damping properties, understanding the behavior and values associated with an individual metamaterial will allow to specify whether the lattice structure is suitable for specific applications, or whether it will fail at a particular stress.

**Figure 5:**
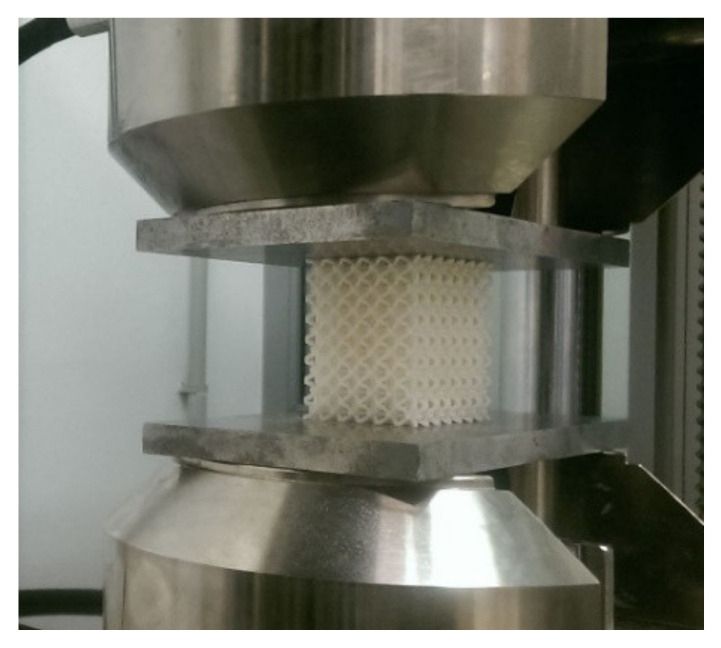
A view of a sample during the process of a compression test.

Uniaxial quasi-static compression tests over the entire range (up to failure) were performed on all investigated grating specimens in the laboratory of the University of Tomas Bata (Zlin, Czech Republic) using a Zwick 1456 testing device (ZwickRoell GmbH & Co. KG, Ulm, Germany) according to the ISO 844 standard [35] at an ambient temperature of 21 °C and a crossbar feed rate of 1 mm/min. The force was measured by the load cell of the testing machine, and the displacement of the machine plate was used to determine the axial strain in the specimen. A view of a specimen during a compression test is shown in Figure 5.

Five samples of the same type (with the same cell size *a* and volume ratio *V_r_*) were tested, and the measured data were recorded. After testing, the stress–strain curves were obtained, while the cross-section areas of each type of the specimens were determined by 3D models in PTC Creo software. The average values of yield strength *σ_Y_* and ultimate strength limit *σ_u_* for each type of structure were calculated. The results were checked for outliers using Grubs’ test criteria.

## 3. Results and discussions

### 3.1. Frequency Dependencies of the Displacement Transmissibility

#### 3.1.1. Effect of Volume Ratio

**Figure 6:**
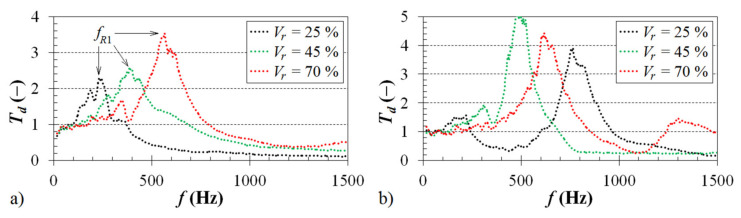
Influence of the sample volume ratio on the displacement transmissibility: (**a**) cell size *a* = 7 mm, inertial mass *m_i_* = 90 g; (**b**) cell size *a* = 10 mm, inertial mass *m_i_* = 0 g.

The volume ratio (or the relative density) of porous materials has a significant influence on their mechanical properties. The mechanical stiffness of porous structures increases with decreasing porosity and pore sizes, which is a typical property of porous materials [36,37,38,39,40]. Figure 6 demonstrates the influence of the volume ratio *V_r_* on the frequency dependencies of the displacement transmissibility *T_d_*. It is evident that the displacement transmissibility increased with an increase in the sample volume ratio (or in its stiffness). For this reason, the vibration damping properties of the investigated ABS samples decreased with an increase in the sample volume ratio, which was reflected in a shift of the first resonance frequency peak position (*f_R_*_1_ ≈ *T_dmax_*) to higher excitation frequencies. This finding is consistent with Equations (3) and (5), when the first resonance frequency generally increases with a decrease in the damping ratio *ζ* and with an increase in the sample stiffness *k*. For example, in the case of the ABS sample, which was produced with a cell size *a* = 7 mm and loaded by an inertial mass *m_i_* = 90 g (see Figure 6a), the first resonance frequency *f_R_*_1_ increased from 226 Hz (for *V_r_* = 25 %) to 585 Hz (for *V_r_* = 70 %). Similarly, the first resonance frequency *f_R_*_1_ increased from 221 Hz (for *V_r_* = 25 %) to 615 Hz (for *V_r_* = 70%) in the case of the unloaded ABS sample (i.e., without the inertial mass) whose structure contained cells of 10 mm in size (see Figure 6b). The measured values of the first resonance frequency for the tested ABS samples, which were produced with three different cell sizes and volume ratios and loaded by three different inertial masses, are presented in Table 2.

#### 3.1.2. Effect of Inertial Mass

**Figure 7:**
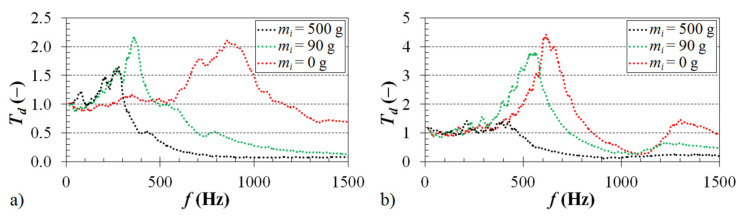
Influence of the inertial mass on the displacement transmissibility: (**a**) cell size *a* = 5 mm, volume ratio *V_r_* = 25%; (**b**) cell size *a* = 10 mm, volume ratio *V_r_* = 70%.

The inertial mass *m_i_*, which is placed on top of the harmonically tested ABS samples (see Figure 4), has a significant influence on the displacement transmissibility, and thus on the vibration damping properties. The effect of the inertial mass on the frequency dependencies of the displacement transmissibility is depicted in Figure 7. It is evident from the displayed frequency dependencies that the inertial mass has a positive effect on the displacement transmissibility, which is reflected in a shift of the first resonance frequency peak position to lower excitation frequencies. As shown in Figure 7a, the first resonance frequency decreased from 848 Hz (for *m_i_* = 0 g) to 266 Hz (for *m_i_* = 500 g) in the case of the ABS sample whose structure was produced with a volume ratio of 25% and a cell size of 5 mm. The first resonance frequency of the ABS sample, which was produced with a volume ratio of 70% and a cell size of 10 mm, exhibited a decrease from 615 Hz (for *m_i_* = 0 g) to 225 Hz (for *m_i_* = 500 g), as demonstrated in Figure 7b. It was confirmed that a higher inertial mass generally leads to a decrease in the natural frequency *ω_n_* (see Equation (4)), and thus to a decrease in the first resonance frequency [41]. It should be noted that the magnitude of inertia load is limited only to the range of elastic deformations of the harmonically loaded ABS samples.

#### 3.1.3. Effect of Material Thickness

**Figure 8:**
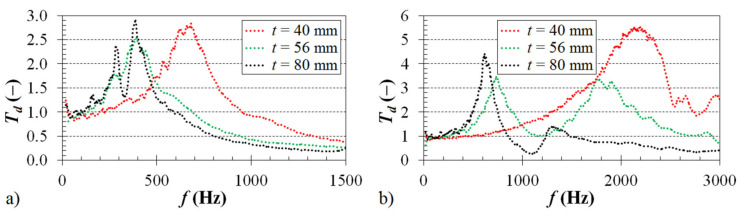
Influence of the material thickness on the displacement transmissibility: (**a**) inertial mass *m_i_* = 90 g, volume ratio *V_r_* = 45%; (**b**) inertial mass *m_i_* = 0 g, volume ratio *V_r_* = 70%.

A material’s ability to dampen mechanical vibration is significantly affected by its thickness *t*, which is proportional to the cell size *a* of the investigated ABS samples. The influence of the thickness on the frequency dependencies of the displacement transmissibility for the samples, which were produced with a volume ratio of 45% and loaded by an inertial mass of 90 g, is shown in Figure 8a.

It is evident that a higher sample thickness generally results in a better ability to dampen mechanical vibration [42,43]. This is accompanied by a decrease of the first resonance frequency (see Table 2) from 682 Hz (for *t* = 40 mm) to 381 Hz (for *t* = 80 mm). This is caused by higher internal friction during the propagation of the mechanical wave through the tested open-porous ABS material structures, and thus by a higher dissipation of the mechanical energy into heat during dynamic loading of these samples. A similar effect of the material’s thickness on the displacement transmissibility was observed for the ABS samples without the inertial mass (*m_i_* = 0 g, Figure 8b), which were manufactured with a volume ratio of 70%. In this case (see Table 2), the first resonance frequency decreased from 2197 Hz (for *t* = 40 mm) to 615 Hz (for *t* = 80 mm).

#### 3.1.4. Influence of Excitation Frequency

As shown in Figure 6, the excitation frequency *f* of mechanical vibrations also has a significant influence on the vibration damping properties of the investigated 3D-printed ABS samples. It is visible that the resonance mechanical vibration (*T_d_* > 1) was generally observed at low excitation frequencies depending on the cell size *a* (or the sample thickness *t*), the volume ratio *V_r_*, and the applied inertial mass *m_i_* under dynamic loading of the tested ABS samples. For example, for the ABS sample with the cell size *a* = 5 mm, the volume ratio *V_r_* = 70%, and without the inertial mass (i.e., *m_i_* = 0 g), the resonance mechanical vibration was achieved at the excitation frequencies *f* < 3150 Hz. In the case of the ABS sample with the cell size *a* = 10 mm, the volume ratio *V_r_* = 25%, and loaded with the inertial mass *m_i_* = 500 g, the resonance mechanical vibration was observed at much lower frequencies (*f* < 210 Hz). Conversely, the damped mechanical vibration (*T_d_* < 1) of the investigated ABS samples was generally observed at higher excitation frequencies (see Figure 6).

### 3.2. Compression Behavior of the ABS 3D-Printed Lattice Structure

Five samples of each type (with the same size *a* and the same volume ratio *V_r_*) were tested, measured data were recorded, and the dependences of loading force and displacement were plotted.

**Figure 9:**
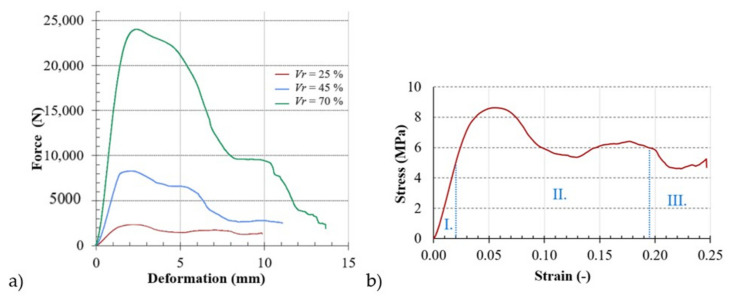
Engineering dependences: (**a**) load vs. displacement of the samples with *a* = 5 mm and *V_r_* = 25%, 45%, and 70%; (**b**) stress vs. strain curve of the sample with *a* = 5 mm and *V_r_* = 25% with regions I. almost linear elastic region, II. constant plateau region, and III. densification area.

As an example, Figure 9a shows the uniaxial compressive responses of BCC lattice structures with the basic cell *a* = 5 mm for three different investigated volume ratios *V_r_* = 25%, 45%, and 70% (see Figure 9a). Based on the plotted load versus displacement, the engineering stress–strain curves were obtained, an example of which (for the specimen with *a* = 5 mm and *V_r_* = 25%) is shown in Figure 9b.

As could be observed on all the plotted curves of the investigated lattice structure, their compressive stress–strain curves show three definite regions/zones: I. a nearly linear elastic region (between zero stress and yield stress), II. constant plateau region (between compressive yield strength and densification strain), and III. densification area (after densification strain), regardless of the rate of “serrate” deformation. The compressive stress increased almost linearly with increasing stress until stress occurred that was accompanied by an overall elastic deformation of the cell walls. From this linear elastic range, the modulus of elasticity was determined according to the methodology [44,45]. As a result of the loading, the cell edge struts stretched and the "cell face" bent [46]. During the next loading, the defects that occurred in the cell shape led to additional local stress concentration and weakened the strength of the wall. As a result, the stress in the non-linear region increased proportion to the relative shortening up to the point of maximum compressive stress.

The recorded values were evaluated by the Grubbs’ outlier test, with one sample showing remote yield strength and ultimate limit tensile strength values. These values were excluded from further processing (this sample was probably made with internal errors, which manifested themselves in low measured values). For each type of sample, average values of the yield strength and ultimate limit strength were calculated, while the values of the measurements were in the range given in Table 3. The standard deviation was not greater than 8% in either case.

**Figure 10:**
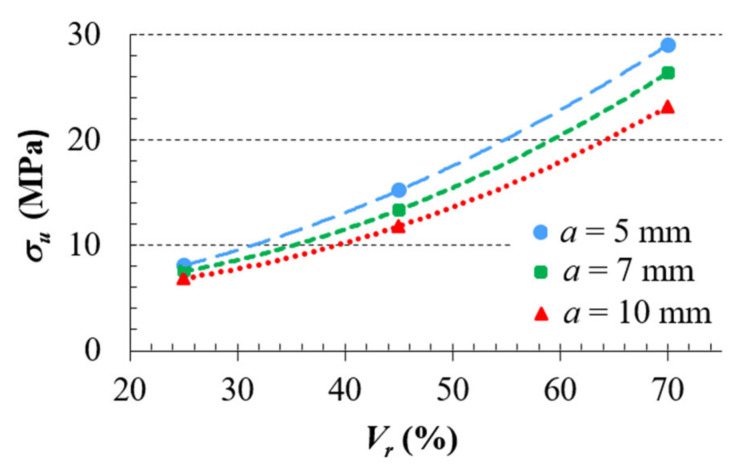
Dependencies of ultimate strength limit on volume ratio *V_r_*.

The average values of the ultimate strength limit were plotted in the graph (as shown in Figure 10) and a trend line, which indicates the dependence of the yield strength on the volume ratio of the sample, was drawn for each cell size; therefore, the behavior of the individual structures under uniaxial compression loading could be identified.

The obtained yield stress *σ_Y_* and ultimate strength limit *σ_u_* values, as well as Young’s modulus *E*, are presented in Table 3.

As the load increased, the cells began to collapse as a result of buckling, crushing, and shearing of the struts. After the breakdown of the first cell, disruptions began to occur in the surrounding cells due to the redistribution of the load. Eventually, the cells lost their integrity and the broken fragments tended to compress, resulting in a significant increase in stress. The stress increased continuously and steeply until it reached the limit stress, which is called compaction. The initial compaction of the cellular metamaterial upon overstress represents the onset of cell wall interactions that increase the compression resistance of the cellular solid [47,48].

**Figure 11:**
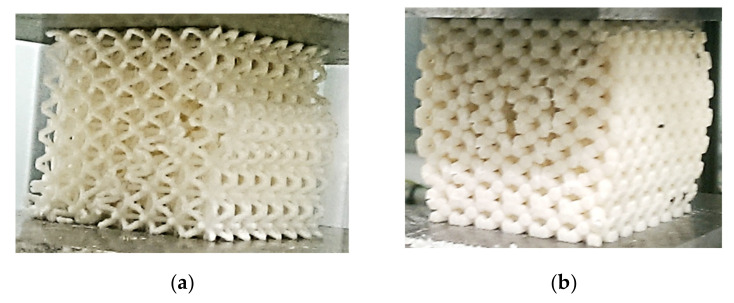
Crack propagation at the lattice structures: (**a**) *a* = 5 mm and *V_r_* = 25%; (**b**) *a* = 7 mm and *V_r_* = 70%.

It was observed that the structures fracture at the weakest pore layer and a crack was propagated in a shear plane, as seen in Figure 11a; although, for the samples with 70% volume ratio and with basic cell sizes of 7 and 10 mm, the cracks propagate symmetrically in height around the center of the faces of the samples (Figure 11b). It can be said that the considered lattice metamaterial (BCC structure made of ABS plastics) has undergone plastic deformation before cracking. Image analysis can be used to predict this, but without a compression test, the preferred path of crack propagation cannot be accurately determined.

**Figure 12:**
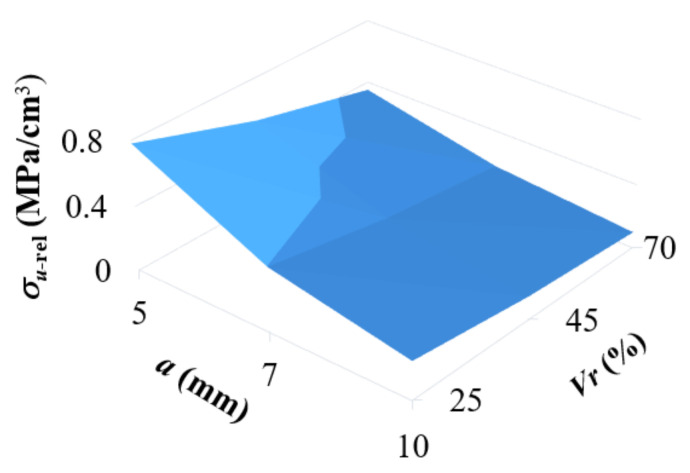
The dependency of relative strength carried by 1 cm^3^ of the consumed Acrylonitrile Butadiene Styrene (ABS) filament on the cell size *a* and the volume ratio *V_r_*.

From a practical point of view, it was interesting to find out which of the studied structures (in terms of combination of cell size and relative volume) can bear the greatest load (to failure) in terms of unit of consumed material (cm^3^ of ABS filament) in 3D printing. From the graph in Figure 12, it can be seen that the most suitable cell for final application in the core of a stress loaded component is the BCC cell with the smallest size and volume ratio; in this case, it is a combination of the size *a* = 5 mm and the volume ratio *V_r_* = 25%.

From the above compression tests, it can be concluded that the mechanical stiffness of the investigated ABS lattice structure, which is characterized by Young’s modulus *E* (see Table 3), generally increased with the increasing volume ratio *V_r_* and the sample thickness *t* and with the decreasing cell size *a*, which is in good agreement with non-destructive dynamic vibration damping tests, when the mechanical stiffness of the ABS lattice samples increased with the increasing first resonance frequency (see Table 2). For these reasons, it can be stated that a good correlation between compression and mechanical vibration damping measurements has been found.

## 4. Conclusions

Lightweight materials with regular cellular structures that exhibit good mechanical properties represent an increasing potential for application not only in biomedicine, but also in many industrial and economic sectors. In the aviation, automotive, and aerospace industries, in addition to using less material to produce the component itself, they have the advantage of requiring less fuel to propel them while weighing less, making these metamaterials environmentally friendly. The fabrication of such structures from metallic and non-metallic materials is now enabled by 3D printing technology. To prevent premature damage and consequent failure of the entire device, the component must meet specific damage tolerance criteria. Therefore, knowledge of the behavior under load is a very important aspect of research before implementation in real conditions. 

The presented work deals with the study of the mechanical vibration damping and compression behavior of a lattice structure made of plastic ABS. The effect of cell size and volume density on the properties of the structure with a body-centered cubic cell fabricated by FDM 3D printing technique was experimentally investigated.

The damping characteristics of lattice structures were not compared with the damping characteristics of uniform cubic structures of the same weight. The same weight of each lattice structure and the uniform structure would lead to different dimensions of individual cube structures. In general, the uniform cube structure is characterized by lower vibration damping properties compared with the investigated lattice structures of the tested ABS material.

The experimental results showed that the ability of the investigated 3D-printed ABS plastic lattice structures to damp mechanical vibration increased with the decreasing volume ratio (or specimen stiffness) and with the increasing cell size (or specimen thickness), inertial mass, and excitation frequency of mechanical vibration, associated with a higher dissipation of mechanical energy into heat and with a shift on the position of the first resonant frequency peak to lower excitation frequencies. It was also found in this study that the main mechanical compressive properties of the BCC lattice structure made of ABS plastics increased with the increase of the volume ratio, as hypothesized, but decreased with the cell size, but the dependency of the ultimate strength on the volume ratio of the metamaterial is not linear. It was observed that the structures break at the weakest pore layer and the crack propagates in a shear plane. However, for the specimens with a volume ratio of 70% and the basic cell sizes of 7 and 10 mm, vertical fracture occurred in the middle of the height.

In this work, from the experimental data, it can be concluded that a correlation was found between the stiffness of the studied ABS plastic lattice samples evaluated by both compressive stress and mechanical vibration damping. Moreover, the vibration damping method is a non-destructive approach to the stiffness of materials, which is an undeniable advantage over compression tests.

## Figures and Tables

**Table 1:** Properties of ABSplus-P430 Ivory material [23].

Property	Symbol	Unit	Value
Ultimate limit of strength	*σ* *_u_*	MPa	37
Absolute extension	Δ*l*	mm	4.4
Young’s modulus	*E*	MPa	1920
Yield strength	*σ* *_Y_*	MPa	37
Poisson’s constant	*ν*	-	0.394
Density	*ρ*	g/cm^3^	1.04

**Table 2:** First resonance frequencies *f_R_*_1_ including their standard deviations in Hz of the tested ABS samples depending on the cell size *a*, the volume ratio *V_r_*, and the inertial mass *m_i_*.

*a* (mm)	*V_r_* (%)	*m_i_* (g)	*m_i_* (g)	*m_i_* (g)
		0	90	500
5	25	848 ± 37	357 ± 13	266 ± 12
	45	1835 ± 62	682 ± 25	269 ± 11
	70	2197 ± 73	775 ± 24	398 ± 14
7	25	470 ± 21	226 ± 9	199 ± 8
	45	587 ± 18	391 ± 12	205 ± 10
	70	721 ± 33	585 ± 22	330 ± 15
10	25	221 ± 10	199 ± 9	183 ± 7
	45	456 ± 14	381 ± 19	188 ± 8
	70	615 ± 24	551 ± 24	225 ± 9

**Table 3:** Average values including their standard deviations of yield strength *σ_Y_*, ultimate limit strength *σ_u_*, and Young’s modulus *E.*

*a*	*V_r_*	*σ_Y_*	*σ_u_*	*E*
(mm)	(%)	(Mpa)	(Mpa)	(Mpa)
5	25	5.0 ± 0.4	8.6 ± 0.4	253 ± 17
	45	7.6 ± 0.4	15.2 ± 0.5	380 ± 20
	70	13.1 ± 0.6	29.0 ± 0.9	653 ± 32
7	25	4.5 ± 0.3	7.5 ± 0.3	227 ± 13
	45	6.9 ± 0.4	13.4 ± 0.5	348 ± 17
	70	12.4 ± 0.5	26.4 ± 0.7	619 ± 26
10	25	4.3 ± 0.2	6.9 ± 0.3	213 ± 12
	45	6.5 ± 0.4	11.8 ± 0.5	325 ± 20
	70	11.7 ± 0.5	23.1 ± 0.6	583 ± 36

## Data Availability

The data presented in this study are available on request from the corresponding author.
